# Driving the effectiveness of public health emergency management strategies through cross-departmental collaboration: Configuration analysis based on 15 cities in China

**DOI:** 10.3389/fpubh.2022.1032576

**Published:** 2022-12-12

**Authors:** Hongmei Wang, Jing Sun, Yinfeng Shi, Tingyue Shen

**Affiliations:** ^1^School of Government, Central University of Finance and Economics, Beijing, China; ^2^Center for China Fiscal Development, Central University of Finance and Economics, Beijing, China

**Keywords:** cross-departmental collaboration, public health, emergency management, COVID-19 pandemic, configuration analysis

## Abstract

**Background:**

Owing to the complexity of and changes associated with modern public health emergencies, cross-departmental collaborative governance is an inevitable choice for ensuring effective emergency management. In the context of emergency management research, the way in which taking full advantage of synergy can be used to enhance the effectiveness of emergency prevention and control approaches is an important issue that must be addressed urgently.

**Methods:**

Combined with China's responses to the management of public health emergencies, in this study, we construct a theoretical analysis framework involving three dimensions: information, organization, and environment. Our proposed framework relies on the fuzzy-set qualitative comparative analysis (fsQCA) method to analyze the mechanisms behind the prevention and control of coronavirus disease 2019 (COVID-19) cases across 15 cities located in typical provinces throughout China and explore the roles of cross-departmental collaboration in the processing of various elements as well as the effects of their combination on the action mechanisms for ensuring the effectiveness of emergency management approaches.

**Findings:**

The results show a significant conditional correlation between the effectiveness of emergency management and the factors affecting cross-departmental coordination. Based on the characteristics of multiple concurrent paths, the driving paths can be classified into four categories: organizational, environmental, environment-balanced, and organization environment-based dual-core categories.

**Conclusions:**

The effectiveness of public health emergency management is the result of multiple factors. Local governments should strengthen the coordination and integration of information, organization, and environment, improve the coordinated system associated with emergency management, promote the “two-wheel drive” of high-quality development as well as accurate prevention and control, explore and perfect the adaptive combinatorial optimization path, and effectively transform the advantages of linking multi-dimensional factors with governance efficiency.

## Introduction

In 2020, the unprecedented coronavirus disease 2019 (COVID-19) outbreak rapidly and spread globally, and it continues to constitute a public health emergency of international concern. Currently, throughout all countries and regions across the globe, the cumulative number of COVID-19 confirmed cases has exceeded 550 million, and the death toll resulting from this pandemic has exceeded six million. In the face of the increasingly severe domestic and international public health security concerns, conventional government-based public health emergency management approaches are no longer effective to manage public health emergencies. The coordination, linkage, and common sharing of multiple subjects across organizational boundaries have become the key to solving complex problems associated with public health emergency management and enhancing the effectiveness of public health emergency management approaches.

The effectiveness of government-based public health emergency management relies on increased levels of integration and collaboration (1). From the perspective of organizational relationships, collaboration involves increased levels of interaction (2). In the context of public health crisis management, interdepartmental collaborative activities can be used to adjust the levels of inter-organizational interdependence, integrate and optimize critical functions, and pool resources, information, and capabilities to form a joint defense taskforce. However, in such contexts, the collaborative process is significantly complicated. Therefore, owing to different interests, objectives of and action criteria as well as imperfect participation channels and mechanisms associated with multiple subjects (3), the phenomena of collaborative relationship dissonance and collaborative behavior deviation still occur occasionally, thereby restricting or even endangering the effectiveness of approaches aimed at managing public health emergencies. The analysis of the responses pertinent to the COVID-19 pandemic established that the improvement of the coordinative mechanisms and the timely development of approaches that can be used to ensure the required capacity of collaborative response are crucial for the prevention and treatment of such epidemics (4). Cross-departmental collaboration results from the concerted efforts of many individuals and elements within and outside organizations. Such elements include event complexity, resource dependence, institutional perfection, and information accessibility (5, 6), all of which play a fundamental role in medical practice, as it pertains to public health emergency management.

Therefore, from the perspective of configuration, based on the collaborative governance theory of public health emergency management, in this study, we construct a theoretical interpretation framework involving three dimensions: information, organization, and environment. Our proposed theoretical analysis framework relies on the COVID-19 prevention and control strategies employed in 15 cities located throughout typical provinces across China. To analyze the samples, we attempt to go beyond the limitations of the associated case studies using the fsQCA social-science method, and we explore the way in which various elements and their combined effects act on the collaborative processes affecting the effectiveness of public health emergency management. Therefore, to provide further insight on government-based public health emergency management mechanisms and their associated paths, in this study, we explain the configuration effects of the related multi-dimensional elements; moreover, we explore the complex interactive nature of multiple elements affecting the effectiveness of public health emergency management strategies, thereby enhancing the effectiveness of public health emergency management systems and their capacity in modern contexts.

## Literature review and analysis framework

### Research on the collaborative governance of public health emergency management strategies

Owing to rapid developments in and the improvement of governance theory, studies on public health emergency management are abundant. At present, scholars mainly study collaborative governance, as it pertains to public health emergency management, from three aspects. The first aspect involves cross-sectoral collaborative governance during public health crises. The root of public health emergency management coordination lies in the particularity of the involved public health emergencies and the inefficiency or ineffectiveness of the associated bureaucratic operations (7). There exists an endogenous contradiction between rigid and sluggish bureaucratic organizations and the high levels of uncertainty associated with public health emergencies; consequently, it is difficult for governmental departments to achieve the expected objectives, as they pertain to public health emergency management. Based on the cross-departmental collaboration model proposed by Bryson and Crosby (8), Comfort and Kapucu confirmed the crucial role of cross-departmental collaboration across organizational boundaries in responding to public health emergencies by analyzing the collaborative relationships established by different organizations through information sharing and the co-construction of resources (9). According to a study conducted by Simo and Bies, cross-departmental collaboration can result in the creation of highly significant public value during responses to major crises, thereby compensating for the deficiency of single department-based responses (10). Facing the most devastating global infectious disease pandemic in a century, Yan and Zhao constructed an analytical framework based on the theory of policy participation using publicly available data on the participation of health practitioners and administrative authorities in policy development as well as implementation, and they verified that collaborative participation-based mechanisms can be used to avoid substantial increases in new cases and deaths during the early stage of the outbreak (4). In addition to public health emergency management under abnormal conditions, some scholars have posited that both horizontal and vertical coordination approaches are necessary for ensuring routine public health emergency management, and collaborative behavior can affect the levels of collaborative efficiency and the associated results (11). To promote the coordination between the normalized peacetime and the sudden emergency mechanisms, it is necessary to further integrate the powers and responsibilities of the relevant departments of public health emergency management to form a joint taskforce focusing on emergency prevention and management (12).

The second aspect involves the processes and factors influencing multi-agent cooperation. Public health emergency management and cross-departmental cooperation are double complexes. Multi-agent collaborative participation is limited by factors at different levels. Studies on this aspect focus on local public health emergency management departments and their relationship with public health institutions by analyzing various characteristics, such as emergency workers population, professional ability, organizational goals, and culture. The similarities of and differences in these characteristics are used to determine the levels of synergy between departments aimed at addressing public health emergencies (13). In such studies, cooperation levels depend on the interaction levels. Scholars have established a three-stage-based risk analysis framework for “perception-understanding-projection” to clarify the interaction processes associated with and characteristics of organizations engaged in the management of public health crisis situations. Further, they argue that communication, information, and technology are the three key dimensions for ensuring the effectiveness of public health emergency management systems (14). By combining the resource dependence theory and the transaction cost theory, a recent study confirmed that the complementarity and sharing of organizational resources is the direct driving force behind multi-departmental synergy, and it effects the stability and sustainability of cooperation (15). Other researchers systematically and comprehensively investigated the Federal Emergency Management Agency's (FEMA) response capabilities in public health emergency situations, and analyzed the effects of organizational systems logic on inter-departmental collaboration. The results demonstrated that institutional factors are essential in the formation and development of partnerships, and that institutionalized arrangements can result in short-term or long-term benefits (16). By analyzing the mechanisms behind the public health emergency management strategies associated with Hurricane Katrina, Waugh and Streib established that the deviation of leadership was a main factor affecting the failure of crisis response. They also established that the dynamic environment as well as the complexity of the events resulted in the liquidity of demand being a crucial reference for collaborative decision-making (17). As a complex systematical approach, emergency coordination is mainly realized through organization, information, resources, planning, and command. Configuring and controlling various elements related to public health emergencies is imperative to ensuring effective coordination between the associated organizations (18). Additionally, some scholars have conducted numerous field investigations and case studies, concluding that prior relationships, trust relationships, power relationships, dependencies, and committed relationships are vital for promoting or hindering cross-departmental collaboration (5, 6, 19). However, owing to their subjectivity, such relational elements cannot be included in the construction of the analysis framework proposed in this study, and therefore, they have not been considered in its construction.

The third aspect involves the effectiveness of collaborative public health emergency management. Government-based public health emergency management approaches focus on the ways in which to rapidly and effectively respond to public health emergencies in a manner that minimizes personal injury and property loss (20). In previous studies, researchers have combined the characteristics of specific crisis events to evaluate and validate the effectiveness of public health emergency management strategies. For example, the World Health Organization relied on multi-dimensional indicators, such as the number of patients/imported cases, the number of deaths/case fatality rate, response time, and early warning time, to evaluate the effectiveness of the responses and strategies used to address the severe acute respiratory syndrome public health crisis (21). Liu et al. innovatively and systematically evaluated the effects of various prevention and control strategies on clustered epidemics from five aspects: epidemic detection, reporting capabilities, precise prevention and control capabilities, public protection capabilities, and the effects of medical treatment (22). Considering the suddenness and urgency of public health emergencies, some scholars obtain public health emergency management-related data from scenario exercises to evaluate the quality of the associated public health emergency prevention and management systems (23). The ideal state of public health emergency management strategies can be achieved only through collaboration, and the associated work effects result only from the coordination and interaction between multiple organizations. However, researchers have demonstrated that different event types and coordination modes have different effects on the effectiveness of public health emergency prevention and control strategies, and cross-departmental coordination may not necessarily result in the enhancement of organizational performance (24). In this regard, Subramaniam et al. used the group behavior model proposed by Robbin to analyze the antecedent conditions of emergency response performance from two levels: organizational resources and personnel structure, thereby providing empirical evidence allowing for further exploration on the ways in which strategies aimed at ensuring the effectiveness of public health emergency prevention and management can be improved (25).

### Construction of the theoretical framework

In the context of emergency management research, public health emergency management strategies have been discussed in-depth. Issues associated with collaborative governance and their effects on public health emergency management strategies have also been explored. Additionally, in such studies, the roles of preliminary inter-departmental coordination as well as the fuzzy relationships between the performance levels of different public health emergency management strategies have been established. However, studies on the use of differentiation pathways to interpret the performance of public health emergency management approaches remain scarce owing to the lack of a combined linkage effect between the elements used in the associated exploratory empirical analyses. In real-world situations, the elements of cooperative behavioral processes associated with public health emergency management are interdependent rather than independent, and their effects on government-based public health emergency management are not similar (18, 26).

**Figure 1:**
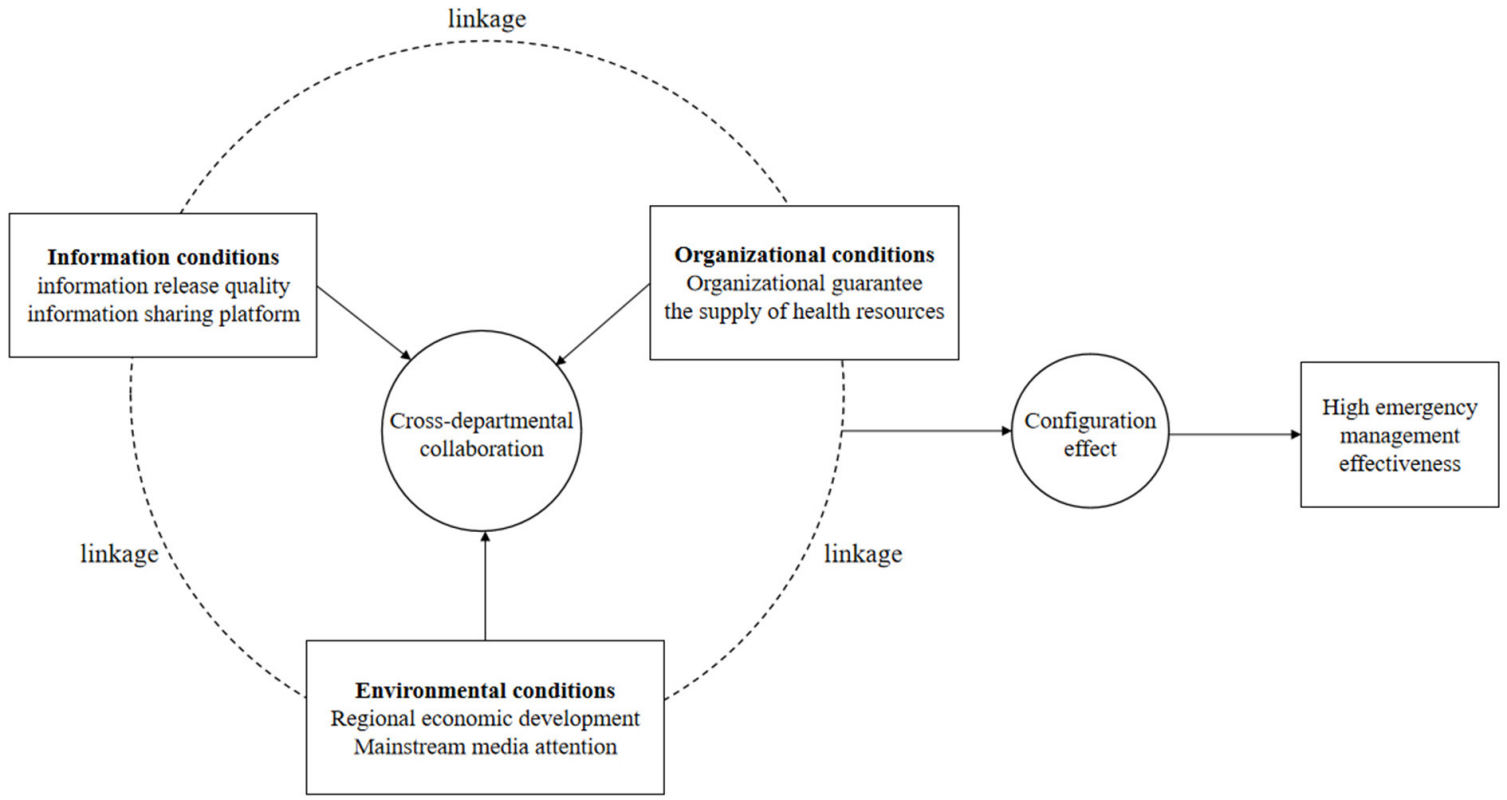
Theoretical analysis framework.

Therefore, on the basis of the theoretical discussion above, this study firstly makes a multidimensional interpretation of the core conditions of technology, organization, and environment in terms of the analysis of the technology-organization-environment (TOE) theoretical framework proposed by Tornatizky and Fleischer (27), combined with the practice of public health management. Among these conditions, organizational elements mainly involve the characteristics and resources of the organization, and environmental elements involve the basic conditions for ensuring cross-departmental collaborative interaction, covering the economic, media, and legal environments, etc. However, in the face of major public health crises, such as COVID-19, the role of information services is essential. Information services reflect the government's technical ability to use big data and they represent the value orientation of serving society through technological infrastructure, an attribute that can be used to effectively demonstrate the government's “political” functions. As a result, the three dimensions of information, organization, and environment should be considered during the selection of condition variables, and the six key antecedent conditions used throughout the process of cross-departmental collaboration should be integrated systematically. In addition, based on the configuration theory and collaborative governance theory, the study emphasizes the interaction and linkage between different elements in the system, and focuses on how cross-departmental collaborative elements drive the effectiveness of public health emergency management through the configuration effect. In conclusion, the theoretical analysis framework of this study (see Figure 1) is constructed, to make an in-depth analysis of the associated relationships revealed the interactive relationships between and the linkage effects of different conditional elements, as they pertain to public health emergency management.

The first element involves information conditions. Specifically, this element includes two secondary conditions: the quality of the information released and the information sharing platform. Information disclosure and sharing is a process, in which emergency subjects collect, process, communicate and utilize the information on risk problems and crisis treatment. It is not only a sharp tool to solve the problem of emergency information islanding, but the basic requirement of government-based public health emergency management, which determines the scientificity and accuracy of emergency management approaches (28). Owing to the advent of the risk society, risk factors in the field of public health have increased significantly, and uncertainties, such as emergency decision-making, risk identification, and social cognition have intensified (29). Integrating and utilizing various government-sourced information tools to ensure the enhanced quality of the information released has become a crucial channel for enhancing the effectiveness of public health emergency management strategies. Both theoretical research and practical experience have shown that (1, 30), to ensure effective responses to major epidemic crises and break away from government-restricted information, the associated committees, emergency management bureaus, transportation bureaus, and other departments have jointly built an information sharing platform based on big data information technology, and formed a carrier for information circulation among the information releasing, using and managing subjects. This platform provides information for reducing the spread of the virus and it provides evidential sources for “evidence-based decision-making” aimed at achieving the long-term and sustainable development of public health emergency management strategies.

The second element involves organizational conditions. Specifically, this element includes two secondary conditions: guaranteed organizational system effectiveness and the effective supply of health care resources. Institutions are norms, and governance mainly involves behavior. The development and evolution of China's public health emergency management system (31) shows that the focus on emergency management coordination has expanded from information and public opinion to plans, teams, and equipment. Moreover, it has expanded from emergency response to the entire processes of prevention, preparation, response, and recovery, thereby gradually forming a strong and necessary prerequisite (16) for ensuring synergy and linkage efficiency among all the parties involved in ensuring effective public health emergency management. Based on the market/general equilibrium theory, the overall insufficiency of health care resources, the shortage of high-quality health care resources, the unbalanced allocation of health care resources, and the untimely supply of such resources are the main challenges experienced during emergencies by medical professionals (32). According to the existing literature, the resource reserve support system, as an important aspect to measure the emergency response capability (33), needs to operate under the mode of peacetime and war combination and synergistic integration of medical and prevention. To avoid public health emergency management failures resulting from the provision of insufficient health resources and to overcome the decreased utilization rates of resources resulting from excessive or extensive emergencies, it is necessary to break the boundaries of departments or regions and optimize the allocation structure of various medical resources, integrate the functions and layout of existing institutions, promote the joint construction and sharing of health resources (9, 34), thus enhancing the overall efficiency of the medical service system.

The third element involves environmental conditions. Specifically, this element includes two secondary conditions: the levels of regional economic development and mainstream media attention levels. Public health emergency management is a vital area, where governments at all levels pay the most attention and consume the most resources. As a foundational attribute, achieving effective public health emergency responses and management strategies requires secure and stable development environment (17, 35). Economic development plays a significant role in achieving enhanced and long-term guarantees for ensuring public health emergency prevention and management. Emergency training and drills, response and management, publicity and education, as well as other process activities are inseparable from the support provided through regional economic development approaches, which can firmly guarantee the safety and health of the public. Throughout “Beijing's,” “Guangzhou's,” and “Zhejiang's” experiences associated with the prevention and control of the COVID-19 pandemic, it can be established that pandemic prevention measures employed in regions with relatively developed economies are highly precise and refined, the resumption of work and production is more orderly, and the levels of coordinated public health emergency response are also significant. Based on the communication theory, mainstream news media on the occurrence, development and epidemic prevention and control process for effective analysis, tracking report all related process and results (36), to supervise and restrict the government behavior, and use their influence to correctly guide public opinion, thereby helping in improving the public's ability to recognize sudden infectious disease epidemics and cultivate personal safety and protection awareness. Through this approach, it is possible to reduce the possibility of the large-scale spread of the virus throughout the population and ensure the effective promotion of public health emergency management strategies aimed at ensuring the prevention of epidemics.

## Research methods and data construction

### Qualitative comparative analysis

In this study, we used the fsQCA social-science method to analyze and explore the multiple mechanisms behind public health emergency management. The fsQCA method, which was first proposed by the sociologist called Ragin, is an analytical approach that integrates both quantitative and qualitative orientations. This method infers multiple concurrent causal relationships between the conditions and results employing set relation analysis. Throughout the processes associated with responding to public health emergencies, the effects of information conditions, organizational conditions, and environmental conditions on the effectiveness of public health emergency management strategies are not entirely independent. However, the related synergistic effects are achieved through linkage and matching. The fsQCA method based on the configuration perspective provides a crucial approach for solving the aforementioned complex causal problem. Through cross-case comparative analysis, complex causal relationships hidden in such cases can be excavated, thereby overcoming limitations associated with conventional regression analyses on the monotonicity of causal relationships and cracking the “namelessness” of micro-large sample data. Additionally, contrary to clearly set qualitative comparative and multi-valued set qualitative comparative analyses, the fsQCA method is significantly advantageous when dealing with complex problems, such as partial membership or membership degree changes. Therefore, in this study, we adopted the fsQCA approach.

### Case selection and data construction

#### Selection of research cases

Currently, the COVID-19 pandemic is a global health crisis never seen in a century. This pandemic has resulted in significant damages to the economic and social development worldwide as well as human life and production. In China, which is a significantly developed country, the outbreak of the COVID-19 pandemic resulted in various departments across the country rapidly launching emergency responses by gathering and integrating forces from all parties. The Chinese government exerted its significant resource mobilization and coordination capabilities, thereby ensuring the effective short-term control of the spread of the pandemic. However, different regions faced different challenges. Owing to the constantly changing public health situations as well as joint prevention and control tasks, the effectiveness levels of various public health emergency management strategies varied significantly. To deeply analyze the various synergistic elements and their associated configuration mechanisms acting on the effectiveness of the public health emergency management strategies, this study is rooted in China's anti-epidemic practices combined with the differentiation of regional development levels and the localization of public health incident responses. According to the methods of theoretical sampling and non-random sampling, we selected the cities significantly affected by the epidemic, and considered the complex situations involving representative provinces, such as Beijing, Hubei, Guangdong, Jiangsu, Sichuan, and Heilongjiang. We also collected and sorted out information related to pandemic prevention and control, as they pertained to the COVID-19 pandemic case data. As listed in Table 1, the selected cases are all centered on the prevention and control of the new crown pneumonia epidemic, covering the two stages of “abnormal emergency” and “normalized prevention and control” throughout Central China, South China, North China, West China, East China, and other regions. The pandemic prevention and control measures as well as the prevention and control effects vary by place, and they generally conform to the principles of “maximum similarity” and “maximum difference,” which can be used to ensure the scientificity and accuracy of the research process.

**Table 1:** Case selection and analysis.

**Prevention and control stage**	**Regional distribution**	**Cities**	**Epidemic prevention and control cases**	**Basic situation of the case**
Abnormal emergency	Central China	Wuhan	The first round of COVID-19	In the epicenter and hardest hit areas of the epidemic, the number of confirmed cases continues to surge, various emergency resources are in serious shortage, and a reference prevention and control experience is lacking in the early stage.
		Huanggang	The first round of COVID-19	
		Xiaogan	The first round of COVID-19	
	South China	Guangzhou	The first round of COVID-19	The situation of “preventing imports from abroad” is severe, with high population density and strong personnel mobility. The pandemic has had several waves since then, and the pressure of prevention and control continues to increase.
		Shenzhen	The first round of COVID-19	
		Foshan	The first round of COVID-19	
Normalized prevention and control	North China	Beijing	Epidemic in Xinfadi	Beijing has a special role as a city. Relying on its previous prevention and control experience, Beijing has adopted “fast, accurate and ruthless” measures to control the epidemic in the shortest possible time, becoming a model for normalized epidemic prevention and control.
	West China	Chengdu	Local clustered outbreaks	Mainly clustered epidemics, with a widespread chain in crowded places, are prone to large-scale spread. However, the overall prevention and control are more accurate and timely.
		Nanchong	Local clustered outbreaks	
		Dazhou	Local clustered outbreaks	
	East China	Nanjing	Lukou Airport Epidemic	The new Delta epidemic at Lukou Airport caused a large-scale spread, and then large-scale clustered epidemics occurred in Nanjing, Yangzhou and other places.
		Yangzhou	Local clustered outbreaks	
		Huaian	Local clustered outbreaks	
	Northeast China	Harbin	Local clustered outbreaks	The combination of imported risk and transmission risk. Local medical institutions and medical staff lack awareness of epidemic prevention and are ignorant of response and management.
		Suihua	New outbreak in Wangkui County	

#### Variable measurement and calibration

##### Outcome variable

The outcome of this study involves the effectiveness of public health emergency management approaches. Government-based public health emergency management approaches involve the rapid response to emergencies, with the main aim being the minimization of personal injury and property damage. The Law of the People's Republic of China on Emergency Response, the National Overall Emergency Response Plan for Public Health Emergencies, and the Regulations on the Management of Public Health Emergencies clearly emphasize on the need to effectively prevent, control, and eliminate public health emergencies. Protecting the public's health, preventing harm, ensuring their safety, and maintaining normal social order are the main objectives of the aforementioned laws and regulations set forth by the government. According to the World Health Organization's measurement standards for the performance of public health crisis prevention and control strategies (21), combined with the particularity and complexity of epidemic prevention and control, the effectiveness of public health emergency management approaches is measured based on two aspects: the effect of medical treatment and the effects of epidemic prevention and control strategies (22). Among them, the effects of medical treatment approaches are measured using various indicators, such as the cure and case fatality rates, and the effects of epidemic prevention and control strategies are measured using indicators, such as epidemic duration and epidemic infection levels. Relevant indicators are mainly derived from the latest epidemic data released by the national and local health commissions between 2020 and 2021.

##### Antecedent condition

First, regarding information conditions, the measurement of information release quality adheres to the provisions of the “Notice of the State Council's Joint Prevention and Control Mechanism for the New Coronary Virus Infection Pneumonia Epidemic on Further Doing a Good Job in the Current New Coronary Pneumonia Epidemic Prevention and Control,” as presented in the epidemic information release mechanism section using the two indicators of information timeliness and information integrity. Among these indicators, the timeliness of information release is assigned depending on whether the epidemic prevention and control press conference is later than the next day. The integrity of information is based on whether the city (prefecture, state, or league) in which the epidemic occurred holds a press conference every day to update the public on epidemic-related information in real-time. The information-sharing platform provides information support for enabling emergency decision-making and ensuring coordinated responses to public health emergencies. By examining the number of local government-based epidemic information sharing platforms, we understand the relationship between information technology and public health crisis responses. Second, regarding organizational conditions, the measurement of organizational system security relies on two indicators: the number of joint documents issued by multiple departments and the number of emergency coordination agencies. Among these indicators, the number of joint documents issued by multiple departments refers to the joint documents issued by local governments, health, disease control, civil affairs, and other departments after the outbreak of the new crown pneumonia. The index data were collected and sorted using government websites, news reports, and research literature. Coordinating the allocation of various key health resources and achieving a balance between the supply and demand of resources is a key measure for ensuring coordinated responses to public health emergencies. The measurement of the supply of health resources relies on three basic indicators (18, 37, 38): health technicians, medical and health institutions, and the number of beds. The relevant indicator data are obtained from the “China Health Statistical Yearbook” and the local statistical bulletin of national economic and social development. Third, regarding environmental conditions, the regional per capita GDP data are collected from the local statistical yearbook as the main measure of the levels of economic development in different regions. The number of news reports reflects the relative importance of a specific issue on the media agenda (39). Therefore, the measurement of news media attention is based on the number of mainstream media reports focusing on epidemic prevention and control strategies, such as Xinhuanet, People's Daily Online, and China News Network.

##### Variable calibration

In fsQCA, each condition (six condition elements in this study) and outcome (emergency management effectiveness) is treated as an independent set, and each case has a membership score in these sets. The original cases must go through a calibration process. The data are converted into fuzzy set of membership numbers. In this study, except for the condition variable of information release quality, which can be assigned “yes” and “no,” all the other variables must be recalibrated. Drawing on the results of existing studies, in this study, we use the direct calibration method (40) to calibrate the antecedent conditions and results based on theoretical and empirical knowledge, combined with the data types of the associated variables. Specifically, 95% and 5% of the sample data are used as the anchor points of the “completely affiliated” and the “completely non-affiliated” attributes, respectively, and the “intersection point” is determined according to the actual situation of the research case. Reasonable adjustments are made according to the realistic problem (see Table 2).

**Table 2:** Calibration of conditions and results.

	**Conditions and Results**	**Calibration anchor**		
		**Completely affiliated**	**Intersection point**	**Completely non-affiliated**
Outcome variable	Emergency management effectiveness	100	94.9860	62.7555
Information conditions	Information release quality	1	/	0
	Information sharing platform	5.6	3	1
Organizational conditions	Organizational system guarantee	13.9	3.2	1.02
	Health resource supply	11.6772	3.0732	1.7211
Environmental conditions	Regional economic development	18.4674	9.9866	3.5235
	Mainstream media attention	986.5	67	0.7

## Data analysis and empirical results

### Necessity analysis of a single condition

Before analyzing the sufficiency of condition type configuration, it is necessary to conduct necessity analysis for the individual conditions individually to determine whether there is a specific condition that constitutes the necessary conditions of the result variable. Therefore, in this study, we adopt fsQCA version 3.0 to analyze the consistency and coverage of each condition variable. Table 3 shows the necessity analysis of a single condition. The results show that the consistency levels for all conditions are lower than 0.9. Therefore, a single condition variable cannot constitute a necessary condition for the result. It is necessary to further explore the combined linkage effect of the three dimensions of information, organization, and environment throughout the cross-departmental collaboration process.

**Table 3:** Necessity analysis results.

**Antecedent variables**	**High-level emergency management effectiveness**	
	**Consistency**	**Coverage**
Information release quality	0.3553	0.8075
~Information release quality	0.6447	0.5329
Information sharing platform	0.6020	0.6961
~Information sharing platform	0.5893	0.7506
Organizational system guarantee	0.5469	0.7364
~Organizational system guarantee	0.6565	0.7237
Health resource supply	0.5172	0.6678
~Health resource supply	0.6423	0.7338
Regional economic development	0.6171	0.7685
~Regional economic development	0.5181	0.6119
Mainstream media attention	0.4258	0.6420
~Mainstream media attention	0.8338	0.8452

“~” indicates absence.

### Adequacy analysis of condition configuration

The adequacy analysis of condition configuration is the core of the fsQCA method, which focuses on analyzing the way in which different configurations of multiple conditions affect the generation of outcome variables. Unlike the necessity analysis of a single condition, the adequacy analysis is based on truth tables rather than fuzzy membership scores. With reference to the results of existing studies, in this study, we set the consistency threshold to 0.8 and the case frequency threshold to 1, thereby constructing and analyzing the truth table to obtain complex solutions, intermediate solutions, and simple solutions. As the intermediate solution only incorporates logical residual terms that are in line with theoretical expectations and practical empirical evidence, the complexity is moderate, and the necessary conditions cannot be eliminated. Therefore, in this study, we mainly use the intermediate solution, which is supplemented by the parsimony solution (41), to determine the core edge conditions of different configurations. Table 4 shows four driving paths resulting in enhanced levels of the effectiveness of public health emergency management approaches, whereby each column represents a possible conditional configuration path. In the different configurations, black circles indicate the presence of the condition, crossed circles indicate the absent of the condition, blank cells represent conditions that do not matter for the solution. Large circles indicate core conditions where the outcome exists and small circles indicate marginal or auxiliary conditions where the outcome exists. In addition, the relevant parameters for each configuration path, such as original coverage, unique coverage, overall solution consistency, and overall solution coverage, are described in Table 4. The study found that the consistency level of both the single solution and the overall solution is higher than that of the standard value, 0.75, and the consistency of the three condition combinations is 1, indicating that the condition combination has a high explanatory power for the outcome variable.

**Table 4:** Configuration analysis of high-level emergency management effectiveness.

**Configuration**	**Organizational type**	**Environmental type**	**Environment-balanced type**	**Organization-environment dual core type**
	**C1**	**C2**	**C3**	**C4**
Information release quality	⊗	⊗	⊗	•
Information sharing platform	⊗	⊗	•	•
Organizational system guarantee	•			•
Health resource supply	⊗	⊗	•	•
Regional economic development		•	•	•
Mainstream media attention	⊗	⊗	⊗	⊗
Consistency	0.9781	1	1	1
Raw Coverage	0.1969	0.1738	0.1651	0.1166
Unique coverage	0.1045	0.0386	0.0464	0.1166
Solution consistency	0.9901			
Solution coverage	0.4413			

Full black circles and crossed-out circles indicate the presence and the absence of causal conditions, respectively. Large circles (• and ⊗) indicate the core conditions, small circles (• and ⊗) indicate the peripheral conditions, and the blank cells represent conditions that do not matter for the solution.

Specifically, Configuration 1 indicates that the existence of organizational institutional safeguards plays a central role. Compared with other conditions, organizational system guarantee is highly important for ensuring the effectiveness of high-level emergency management approaches, and this attribute can be used to independently constitute a sufficient condition to explain the results. Under this condition configuration, when the organizational system guarantee exists, other conditions are insignificant for ensuring the effectiveness of high-level emergency management strategies. Therefore, this path can be summarized as “organizational type.” This means that effective organizational system guarantee can break through the constraints of information, environment, and other conditions affecting the ability for local governments to improve the effectiveness of public health emergency management approaches. The effectiveness of COVID-19 responses cannot be separated from the full play of emergency response capacity, which is closely related to institutions. Francis Fukuyama's conclusion on the determinants of epidemic effectiveness also proves the decisive role of institutional factors. The results of Configuration 1 further support the perspective that in the institutional guarantee, to constantly improve the important premise, responses to outbreak crises can fully ensure that the strong ability of information and environmental aspects of the internal and external barrier are broken, and the public's trust in the government can be improved to inspire strong political cohesion and produce high epidemic control results.

Configuration 2 shows that the level of regional economic development plays a central role. Regardless of whether other conditions are complete, a high level of emergency management can be achieved when a high level of regional economic development is maintained. This path is based on the level of regional economic development as the core condition; thus, it can be summarized as the “environmental type.” The level of regional economic development directly or indirectly determines the demand for public health services, and it affects the awareness and ability of public participation (23), which is extremely important for demand-oriented public health emergency prevention and control. Public health emergency management covers the entire process of training and exercise, response and disposal, publicity and education, among other activities. These prevention and control behaviors cannot be supported by economic development. For example, in the early stage of the COVID-19 pandemic outbreak, Huai'an lagged behind other regions in terms of epidemic information release, the joint publication of departments, the establishment of coordination institutions, and the attention of mainstream media. However, relying on the relatively stable local economic development contexts, Huai'an has continuously learned from the prevention and control experiences as well as lessons from the surrounding cities. This has resulted in the provision of the necessary medical and health care materials and equipment; moreover, it has ensured technical support for the response to epidemic, the timely control of the spread of the epidemic, and the achievement of phased prevention and control results.

Configuration 3 shows that cities with a higher level of regional economic development have a higher level of emergency management effectiveness in the context of multiple information-sharing platforms, and they have an adequate supply of health care resources. Among them, the regional economic development level plays a central role, and the information sharing platform as well as the supply of health resources plays a key supporting role. Epidemic prevention and control work in tandem with economic and social development. Economic and social development provides crucial support for epidemic prevention and control, while epidemic prevention and control is a necessary prerequisite for ensuring steady economic and social development. However, when public health emergencies occur in economically developed cities, such as Beijing, Shenzhen, Nanjing, and Wuhan, the speed of transmission, the difficulty of management and control, and the degree of harm are much higher than those in other regions, and the public security situation is even more severe. It is necessary to rapidly integrate and gather enough resources as well as materials and simultaneously build a parallel and multi-point information sharing platform to ensure the smooth and orderly flow of various epidemic-related information to achieve a balanced linkage between resource integration and allocation as well as information co-construction and sharing. This approach can ensure the achievement of accurate prevention and control strategies that can be run efficiently. As all the three conditions of information, technology, and environment must be coordinated and cooperated to ensure their effective functionality, in this study, we summarize this path as the “environment-balanced type.”

Configuration 4 shows that cities with strong institutional advantages and economic support produce high-level emergency management effects if the quality of epidemic information release is high. The information sharing platform is relatively complete, and the public health resources are raised and dispatched reasonably and efficiently. Response to public health emergencies is a complex and systematic project. While strictly implementing territorial responsibilities and main responsibilities, local governments should also actively integrate and coordinate resource elements, such as multi-departmental, multi-level, multi-field, and multi-regional resources, and they must engage such elements fully. Regarding the synergistic effect, because this path contains two core conditions: organizational condition and environmental condition, it can be summarized as the “organization-environment dual core type.” Since the outbreak of the epidemic, the prevention and control situation has been complicated and severe, and the new crown virus has been mutating constantly, thereby resulting in the overlapping of abnormal emergency response and normalized prevention and control. This attribute has resulted in new and higher requirements for urban emergency management. In this regard, Guangzhou City is actively exploring and summarizing the experience of anti-epidemic, thereby providing a satisfactory governance model for ensuring the city's precise prevention and control strategies. For example, to better serve the overall situation of the city's epidemic prevention and control strategies while doing a solid job of normalizing social security for epidemic prevention and control, the Guangzhou Municipal Bureau of Human Resources and Social Security, the Medical Insurance Bureau, and the Taxation Bureau jointly issued the “Regarding Social Insurance Payments During the Prevention and Control of the Novel Coronavirus Pneumonia” and “the Notice on the Work of Claims and Benefits,” and they steadily promoted the normalized prevention and control strategy. Additionally, Guangzhou's municipal government information sharing services have been at the forefront of the country, providing services, such as the establishment of information sharing platforms, including the Guangzhou Municipal Government Information Sharing Platform, the Smart Guangzhou Space-Time Information Cloud Platform, and the Guangzhou Deployment Command Screen to provide information for the acquisition, disposal, and the use of epidemic information channels, thereby enabling the accurate determination of the supply and demand of various resources and effectively improving the utilization efficiency of urban health resources.

### Robustness check

In this study, we conduct a robustness test of the antecedent configuration of high-level emergency management effectiveness. First, keeping other conditions constant, increasing the consistency level from 0.8 to 0.85 produces the same configuration results. Second, considering that the sample cases, such as Northeast China, may have significantly different results from those of other cities in other regions in terms of resource endowment, economic structure, and location conditions, etc., after deleting the two case cities of Harbin and Suihua, except for a slight change in the level of consistency, similar results and nearly consistent conclusions can still be obtained (see Table 5). Therefore, the results of this study are robust.

**Table 5:** Robustness test results for excluding sample cases.

**Configuration**	**High-level emergency management effectiveness**			
	**C1**	**C2**	**C3**	**C4**
Information release quality	⊗	⊗	⊗	•
Information sharing platform	⊗	⊗	•	•
Organizational system guarantee	•			•
Health resource supply	⊗	⊗	•	•
Regional economic development		•	•	•
Mainstream media attention	⊗	⊗	⊗	⊗
Consistency	0.9706	1	1	1
Raw coverage	0.1632	0.1780	0.1695	0.1310
Unique coverage	0.0766	0.0434	0.0522	0.1310
Solution consistency	0.9888			
Solution coverage	0.4378			

The meaning of the symbols in this table has the same meaning as in Table 4.

## Conclusions and recommendations

From the perspective of configuration, in this study, we discuss the complex causal mechanisms that act on the elements of collaborative governance processes aimed at driving the effectiveness of public health emergency management strategies. First, a single synergistic condition element cannot constitute a necessary condition for achieving high levels of emergency management effectiveness, which deepens the study results on the correlation analysis of internal and external factors, such as information, organization, and environment, affecting the effectiveness of emergency prevention and management strategies. Second, there are four configuration paths with high levels of emergency management effectiveness. Previous studies have shown a fuzzy correlation between cross-departmental collaboration and the effectiveness of public health emergency management strategies. In this study, we verify the complex causal relationships between the two, and we establish different synergies. The combined substitution effect between elements provides a crucial practical reference for accurately improving the effectiveness of emergency prevention and control. Third, the cases covered by the configuration with high levels of emergency management effects include economically developed areas, such as Guangzhou and Foshan, as well as economically underdeveloped cities, such as Nanchong and Huai'an, which have better basic conditions and relatively in place institutional guarantees. This demonstrates that emergency management and the achievement of the effects cannot rely only on one aspect or field to play a role. However, the comprehensive results of the linkage and matching of multi-dimensional elements, which have the characteristics of different paths and multiple concurrencies, are significant.

To further exert cross-departmental synergy and comprehensively enhance the effectiveness of public health emergency management, in this study, we present the following suggestions based on the research conclusions. Above all, we strengthen the coordination and integration of information, organization, and environment. Public health emergencies are social phenomena that do not depend on people's intentions, as they pertain to economic and social development. They inevitably involve multi-level, multi-sector, multi-field, and other multi-dimensional problems. Local governments should activation of various factors in the process of collaborative governance based on multi-subject coordination, to promote the integration of the elements to realize the maximization of public health emergency management effectiveness. Therefore, local governments must start from a “holistic” perspective, strengthen the linkage and matching of multi-dimensional factors such as information sharing, system support, and resource supply, and take multiple measures to improve the collaborative emergency response capacity. Furthermore, it is important to attach great importance to the central role of the emergency management coordination system and regional economic development level. Even when other conditions are relatively limited, the construction of linked emergency management system and regional economic development are also ways to improve the effectiveness of government emergency management. On the one hand, for cities with relatively weak institutional construction, the primary task is to accelerate the implementation of joint prevention and control policies, build a multilateral and multidimensional rule system, give full play to the comparative advantages and linkage effects of departments, institutionalize and sustain them, and break through the temporary dilemma of the collaborative emergency response system. On the other hand, we will give full play to the role of market mechanism as a policy tool, actively establish cooperative partnerships with local enterprises, promote the resumption of work and production in an orderly manner, and coordinate the implementation of the tasks of releasing consumption potential and expanding effective investment, so as to provide a safe and stable development environment for joint prevention and control. Finally, it is important to explore and improve the adaptive combination optimization path. Different risk level areas of resources endowment and economic structures, with varying location conditions. All the elements of interactive collaborative governance processes and linkage modes are not the same, and the applicable collaborative strategies also have different focuses. For example, for regions with a high level of economic development, more attention should be paid to the ability of information sharing and resource supply, to accurately and effectively cope with the complex and volatile public health security situation. With the simultaneous improvement of institutional construction and economic development level, various government information tools should be further used to build information sharing platforms and carry out dynamic supervision, to maximize the use of resources inside and outside the organization to enhance urban resilience. Therefore, government management practices should be combined with their social development level and the local part of the management pattern, thereby allowing for the constant optimization of the link between different elements and adaptations. Moreover, exploring the characteristic configuration paths to improve the effectiveness of emergency management depending on local conditions, as well as effectively transform the advantages of linking multi-dimensional factors with governance efficiency.

In this study, we systematically explored the complex mechanisms between cross-departmental collaboration and emergency management effectiveness; moreover, we extended our study beyond the monotonicity analysis of causality found in existing quantitative studies on this subject. We also identified multiple configuration paths that produce high levels of emergency management effectiveness, which also provided new ideas and methods for collaborative governance of emergency management. This study also provides additionally feasible exploration paths for emergency management practice of different countries, and further promotes the transformation of synergistic advantages into governance effectiveness, so as to jointly respond to global public health crises. However, similar to most previous studies, this study inevitably has some shortcomings. This study focuses on the analysis of multi-agent collaborative process elements, and the other factors affecting the effectiveness of emergency management, such as network relations and network structure, have not been considered fully. Owing to the continuous development of global epidemic prevention and control strategies, the existing sample cases may have specific limitations. Future studies can attempt to collect more valuable case data to further improve the universality and scientific nature of research conclusions.

## Data availability statement

The original contributions presented in the study are included in the article/supplementary material, further inquiries can be directed to the corresponding author.

## Author contributions

HW: study conception, design, and writing. JS: study design, empirical analysis, and writing. YS: study design, acquisition of data, and writing. TS: review and editing. All authors contributed to manuscript revision, read, and approved the submitted.
